# The Development of the Mental Representations of the Magnitude of Fractions

**DOI:** 10.1371/journal.pone.0080016

**Published:** 2013-11-13

**Authors:** Florence C. Gabriel, Denes Szucs, Alain Content

**Affiliations:** 1 Centre for Neuroscience in Education, Department of Experimental Psychology, University of Cambridge, Cambridge, United Kingdom; 2 Laboratoire Cognition, Langage et Développement, Faculté des Sciences Psychologiques et de l’Education, Université Libre de Bruxelles, Brussels, Belgium; The University of Western Ontario, Canada

## Abstract

We investigated the development of the mental representation of the magnitude of fractions during the initial stages of fraction learning in grade 5, 6 and 7 children as well as in adults. We examined the activation of global fraction magnitude in a numerical comparison task and a matching task. There were global distance effects in the comparison task, but not in the matching task. This suggests that the activation of the global magnitude representation of fractions is not automatic in all tasks involving magnitude judgments. The slope of the global distance effect increased during early fraction learning and declined by adulthood, demonstrating that the development of the fraction global distance effect differs from that of the integer distance effect.

## Introduction

 Common fractions (hereafter, fractions) denote rational number quantities by the ratio of two whole (integer) numbers (e.g. 1/3). Fractions are theoretically crucial as they depend upon a deeper understanding than natural numbers [1]. They are also fundamental in mathematics education as they play a major role in higher-level mathematics topics such as probabilities and algebra. However, fractions represent considerable difficulty for children studying arithmetic. The difficulty in comprehending fractions may seem surprising as several theorists assume that the understanding of magnitude and ratios is evolutionarily hardwired [2,3]. In fact, evidence is still controversial whether adults represent the global magnitude of fractions at all, or rather, they only rely on integer components when interpreting fractions [4-7]. Further, in contrast to the theoretical and practical significance of fraction learning, to date only a couple of studies examined the mental representations of fractions in children [8,9]. Here, our objective was to gain further detailed understanding of how the representation of fractions changes with development right during the school years when fraction knowledge is just consolidating. To this end we tracked global magnitude activation in grade 5, 6 and 7 children (10- to 12-year-olds) as well as in adults in a number comparison task and in a number matching task, and studied whether global fraction magnitude is activated automatically or not.

Some authors suggest that we have an evolutionary ancient cognitive system that allows us to approximate ratios. This system designed for proportional understanding would be present in monkeys [3], preverbal children [10] and young children [11]. So children would be able to process ratios before receiving formal instruction on fractions. From this angle it is striking that fractions are one of the most difficult topics of early mathematics education to grasp [12,13]. Children experience difficulties to apprehend the global magnitude of fractions (i.e. their real value) and do not seem to have an appropriate representation of the quantity they symbolize. For example, most grade 5 children fail to place a fraction on a graduated number line [14] and struggle when asked to order fractions [15]. Pupils often process numerators and denominators as being two independent whole (natural) numbers and then apply procedures that can only been used with whole numbers [16,17]. This phenomenon is the “whole number bias” [18]. Consequently, pupils make mistakes such as 1/4 + 1/2 = 2/6 when they use erroneous componential strategies to solve this type of problem. 

The above difficulties are directly related to the question of how fractions are represented in the human mind. Currently it is debated whether fractions are mentally represented by maintaining a separate representation of whole number components and/or by having a representation of the global magnitude of the fraction which may be based on the evolutionarily grounded magnitude representation. Before discussing this controversy, it is necessary to mention that evidence suggests that the magnitude of standalone integers (which provide the components of fraction notation, like '3' and '4' in '3/4') is most probably represented in a non-verbal approximate format [19]. The most robust signature of this system is its ratio sensitivity which means that number discrimination is generally subject to Weber's Law, i.e. the ratio between the to-be-compared numbers determines discrimination performance [19]. A consequence of ratio sensitivity is the so-called symbolic numerical distance effect which means that closer numbers are harder (slower and more error prone) to compare than further away numbers (e.g. comparing 1 vs. 4 is faster and more accurate than comparing 1 vs. 2). The symbolic distance effect has been shown in children in overt number comparison [20,21] and in the numerical Stroop paradigm which does not require explicit number magnitude analysis [22,23]. Further, EEG studies have demonstrated that magnitude information represented by single digits is accessed as fast in young children as in adults even if no explicit magnitude processing is required and when perceptual properties of stimuli are perfectly balanced [23]. The above findings suggest that the magnitude analysis of single digits is fast and automatic even in young children. Hence, theoretically it is very likely that the processing of individual digits as fraction components can be similarly fast and automatic.

Studies on the mental representation of fractions used the distance effects between the to-be-compared fraction magnitudes and between their components as indices of global or componential processes. In a comparison task, Bonato and colleagues showed distance effects only between fraction components [4]. Hence they concluded that participants relied on the magnitude of components only. Kallai and Tzelgov [5] used a numerical comparison task with fractions and integers and a physical size comparison task assessing the automaticity of fraction processing. Their results showed that when fractions were compared to natural numbers, participants relied first on the magnitude of components and then accessed the global magnitude. When participants compared a proper fraction (i.e. smaller than 1) to a natural number, the distance effect denoted the activation of what the authors called a generalized fraction (i.e. an entity smaller than one with a constant value). However, when pairs of fractions were compared, participants mostly relied on componential strategies. In contrast, other studies argued that not only componential (i.e. the separate magnitudes of the numerator and denominator) but also global fraction magnitude (i.e. the overall magnitude of the whole fraction) is represented. Meert and collaborators [6,7] combined the comparison task with a priming manipulation. A comparison between two fractions served as prime for a consecutive comparison between two natural numbers. Results showed that both the magnitude of components and the global magnitude of fractions were accessed. Another recent study using a comparison paradigm showed that educated adults can mentally represent the global magnitude of fractions [24]. Ischebeck, Schocke & Delazer [25] and Jacob and Nieder [26] used functional magnetic resonance imaging (fMRI) in comparison and neural adaptation tasks, respectively. The global magnitude of fractions modulated the activity of areas of the intraparietal sulcus in both studies. These results suggest that within the intraparietal sulcus, a fraction might be represented by its global magnitude, rather than by the magnitudes of its numerator and denominator. In summary, evidence from adults is controversial; there is support for both the componential and global magnitude representation view of fractions.

While to date only about a handful of studies investigated fraction processing in adults, to our knowledge, only two studies have so far been conducted on the mental representation of fractions in children. In the first study, Meert and colleagues [8] tested grade 5 and grade 7 Belgian children in a comparison task. The influence of the congruity between the global magnitude of a fraction and the magnitude of its components on fraction processing was assessed. Pupils had to compare fractions with common denominators or common numerators. For pairs of fractions with common denominators (e.g. 3/8_7/8), the magnitude of the numerator is always congruent with the global magnitude of the fraction (e.g. in this case, 7 is larger than 3 and 7/8 is larger than 3/8). In this situation, strategies based on numerator magnitude processing only would be successful. However, for pairs of fractions with common numerators (e.g. 2/3_2/7), the magnitude of the denominator is incongruent with the global magnitude of the fraction (e.g. in this case, 7 is larger than 3, but 2/3 is larger than 2/7). In this situation, strategies based on holistic processing might be beneficial in terms of cognitive cost as there is an incongruity between the magnitude of the denominator and the global magnitude. Numerical distance effects were then tested in a comparison task involving fractions. Additionally, a priming paradigm was used. The comparison of fractions preceded a comparison of natural numbers. The natural numbers of interest were components used in the primed fractions (e.g. 2/3_2/7 primed 3_7). If participants access the global magnitude for fractions with common numerators, interference for the comparison of natural numbers should be observed as participants first had to inhibit the response due to the incongruency for the comparison of fractions. Result showed that pupils used both the global magnitude of fractions and the magnitude of components. Hence, it was concluded that a hybrid (global and componential) representation of fraction magnitude exists. Such conclusions were drawn from the second study where children were asked to either place a fraction on a number line or translate a given position on a number line into a fraction [9]. When a fraction was given to map onto a number line, children used holistic strategies; but when a spatial cue was translated into a numerical fraction, children used componential strategies.

In the current study we examined the development of the activation of the mental representation of global fraction magnitude. Going beyond previous studies we used two fraction processing tasks conceptually tapping into different levels of global magnitude processing, a number comparison task and a number matching (same/different judgement) task. Both tasks are robust and reliable measures of number processing [27]. In the number comparison task (hereafter: comparison task) participants selected the numerically larger of two numbers. In the number matching task (hereafter: matching task), participants decided whether two fractions equalled each other numerically, or were numerically different.

**Figure 1:**
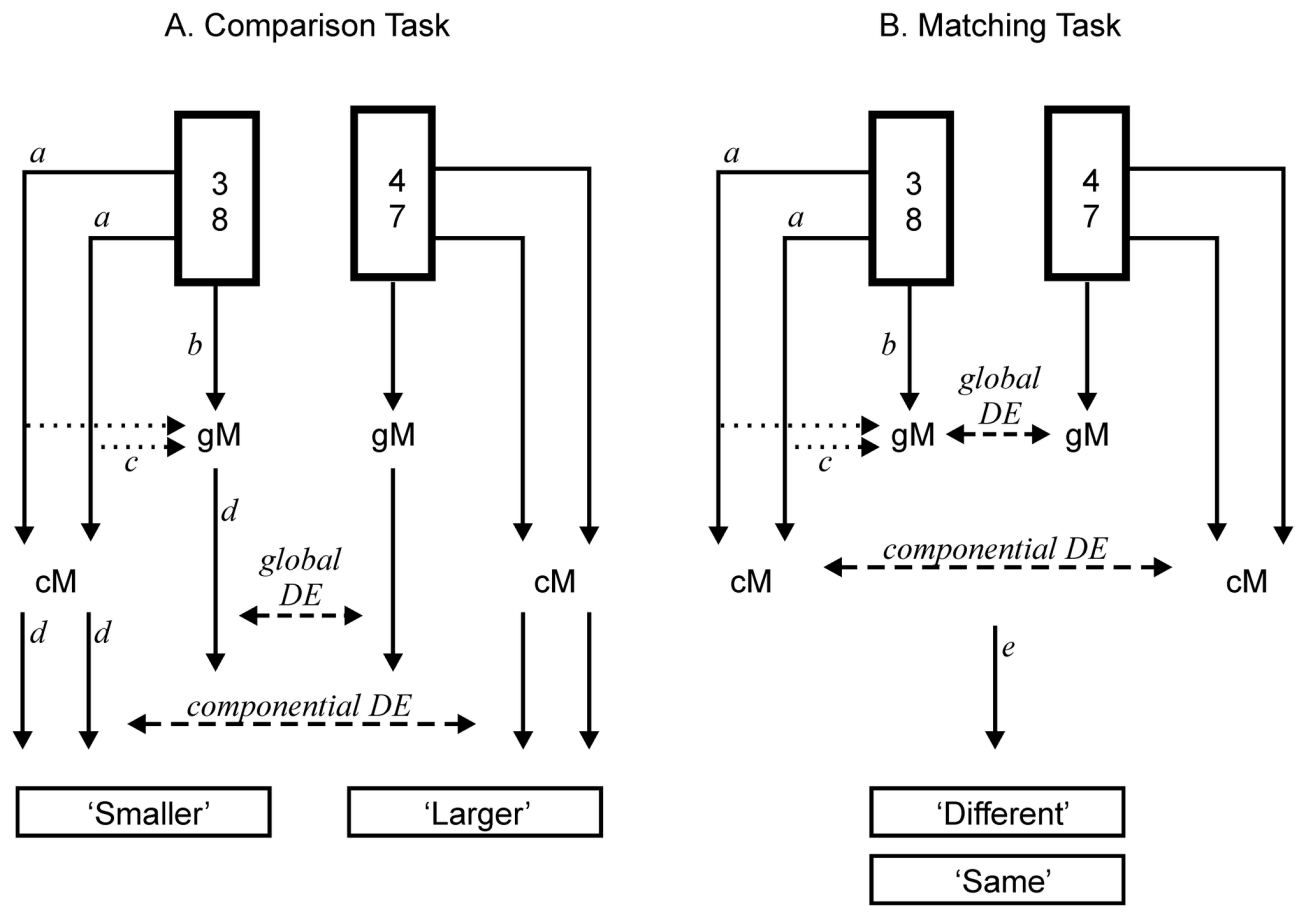
Potential theoretical models of the two experimental tasks. This figure shows models of the Comparison (A) and the Matching (B) tasks. The to-be-compared fraction pairs are shown in the thick boxes (3/8 vs. 4/7). [cM] = Componential magnitude representation, that is, the representation of individual digits, like that of '3'. [gM] = The global magnitude representation of a fraction. Arrows labeled [a] = activation of componential magnitude representation. Arrows labeled [b] = direct activation of global magnitude representation. Arrows labeled [c] = indirect activation of global magnitude from componential magnitude representations. Arrows labeled [d] = Activation of response labels associated with magnitudes. Arrow labeled [e] = activation of response labels in the matching task. In the comparison task distance effects result from the differential strength of stimulus/response associations (association between cM/gM and arrows with [c]). In the matching task distance effects result from the representational overlap between magnitude representations.

Potential theoretical models of the two experimental tasks are shown in Figure **1**. In the comparison task componential magnitudes (‘cM’ in Figure 1) are likely activated (arrows with ‘a’). Global magnitudes (‘gM’) can potentially get activated directly by the whole fraction (‘b’) or after componential magnitudes are activated (‘c’). Finally, magnitude analysis outcomes are linked to response labels and response processes (‘d’ and ‘e’). A major difference between the tasks is that in the comparison task participants make an explicit relative global magnitude judgement between the two fractions and label them appropriately as 'smaller' or 'larger'. Hence, as it is typically the case in psychophysical comparison tasks, a global distance effect can be expected with high probability. However, these effects may only reflect general comparison processes rather than representational properties as shown for example by Holloway and Ansari [28]. In contrast in the matching task participants do not have to make an explicit relative global magnitude judgement and do not have to label fractions by their relative global magnitude, or even extract the global magnitude information. Moreover, all analysis-relevant different fraction pairs are assigned to the same response option (‘Different’). First, this response option is linked to the fraction pair rather than to a single fraction. Second, the 'Different' response option is unlikely to be uniquely associated with any of the individual fractions in a pair. Hence, stimulus-response associations with single fractions most probably do not manifest in performance measures. Hence, the matching task poses a more stringent test for a magnitude representation related global distance effect than the comparison task and potentially appearing global distance effects could be attributed to the manifestation of representational overlap rather than to stimulus-response associations.

The above account is compatible with the view of Van Opstal and Verguts ([29]; see also 30) who suggested that the comparison distance effect is the consequence of a decision process based on the strength of stimulus-response associations rather than a direct expression of accessing number representations. As shown in Figure 1, according to this suggestion, the global distance effects in the comparison task represents the relative strength of response associations linked to both numbers (the arrow labelled 'global DE'). Hence, the global distance effect would not be a pure measure of the magnitude representation in comparison tasks; rather, it would be confounded by decision processes. In contrast, Van Opstal and Verguts [29] argued that the distance effect emanating in same/different judgement tasks is a consequence of accessing number representations. Overall, both above accounts suggest that the matching task is a more stringent test of global magnitude activation than the comparison task. In fact, it is a significant empirical question whether and how the global distance effect is different in the two tasks. Most probably, a stronger distance effect can be expected in the comparison than in the matching task and the difference between the tasks could characterize the difference between the distance effect related to stimulus-response associations (comparison task) and representational overlap (matching task).

A significant difference between integers (symbolic digits) and fractions is that the digit-to- (global) magnitude link is overlearnt already by the end of grade 1 as demonstrated by behavioural and Stroop congruency effects [21] and task-irrelevant symbolic distance effects [31]. In contrast, global magnitude information is usually implicit in fraction notation and relative magnitude relations are often far from evident especially when several different fractions are presented (90 in our experiment). As shown in Figure 1, the magnitude of the individual components of fractions can be expected to get activated (arrows labelled 'a') and the global magnitude may also get activated (arrows labelled ‘b’ and 'c'). However, it is an empirical question whether this global magnitude is activated and is used for example for associating with response labels in certain task contexts. Hence, while a global distance effect in the comparison task may only be a marker of stimulus-response associations [29], it is also a significant question whether this global magnitude activation happens at all in a task-relevant manner (that is, it cannot be taken for granted as in the case of integers). As reviewed above, previous fraction comparison studies provide controversial evidence. 

Our intention was to study the initial stages of fraction knowledge consolidation. Formal fraction instruction starts at grade 4 in the French Community of Belgium where the testing was carried out. As grade 4 pupils still make a large number of errors in fraction tasks we decided to test from grade 5 onwards in order to get stable results. Therefore, grade 5, 6 and 7 (10 to 12 year-old) Belgian pupils as well as adults took part in the study. Involving adults allowed defining the mature state of the representation of fractions and using it for comparison with children. First, we aimed to see whether the comparison and matching tasks show dissociation in any of the age groups. As outlined above, global distance effects in the comparison task can be attributed to stimulus/response associations. However, their mere presence can suggest that global magnitude is activated at least in a task-relevant manner. In contrast, global distance effects in the matching task would appear in the absence of an explicit (magnitude) comparison task. Hence, potential global distance effects could be attributed to the manifestation of a global fraction magnitude representation. Second, we studied the development of global distance effects. Sekuler and Mierkiewicz demonstrated that the slope of the distance effect is decreasing with age in single-digit symbolic integer magnitude discrimination [21]. Meert and colleagues did not report slope changes with grade 5 and 7 children in comparison task [8]. Here we aimed to replicate those results with both the comparison and matching tasks with 3 child groups and one adult group. Including the adult group also allowed for tracking potential long-term changes in the slope of the global distance effect. We also investigated the functional significance of global distance effects in relation to 'real-life' measures of mathematical performance with fractions. Children solved paper and pencil tests besides the experimental tasks. The items were based on tasks used in previous studies which measured children’s understanding of fractions [13,14]. There were five different tasks: estimation task, comparison task, graduated number lines, arithmetic operations and simplification of fractions. In the estimation task, pupils were asked to place a fraction on a non-graduated number line. Estimation has been identified as one of the most important aspect of student’s initial learning of fractions [32]. The comparison task was similar to the experimental task without any time pressure. Graduated number lines allowed us to measure how children represent quantities symbolized by fractions. In the arithmetic task, pupils were asked to solve addition, subtraction and multiplication of fractions. In the simplification task, they had to reduce fractions to their lowest terms by dividing both the numerator and denominator by their greatest common divisor. Test performance was then related to global distance effect measures.

## Methods

### Participants

Initially 117 children took part in our study: 45 grade 5, 35 grade 6 and 37 grade 7 children. Twenty-five grade 5 children, twelve grade 6 children and seven grade 7 children were excluded from the sample because their mean score was below 60% in one or several of the experimental tasks. Therefore, the final sample was composed of 73 children: 20 grade 5 children (mean age: 10 years 5 months old, 5 girls), 23 grade 6 children (mean age: 11 years 6 months old, 10 girls), 30 grade 7 (mean age: 12 years 4 months old, 16 girls). All children came from the same racially diverse population and were from middle socio-economic background. Fifteen young adults were also tested (8 females, 3 left-handed). They were all graduate students (mean number of years of graduate education = 4). The mean age was 24 years (range 22-29). For children, written consent was obtained from parents and head teachers. Adult participants signed a consent form. The study received ethical permission from the Ethics Committee of the Faculty of psychology and education sciences at the Université Libre de Bruxelles.

### Stimuli

#### Paper and pencil tasks

Children's knowledge of fractions were assessed by a paper and pencil task (see Information S1). There were five tasks: estimation, comparison, graduated number lines, arithmetic operations and simplification. The estimation task involved 4 items for which pupils were asked to place a fraction on a non-graduated number line going from 0 to 1. One point was given if they placed the mark at a point situated within 1cm of the right location, and no point was attributed if the mark was further away. In the comparison task, children chose which of two fractions was larger. There were 16 pairs of fractions with different features: same denominators, same numerators, no common components and comparing a fraction to the unit (e.g. 1/2_4/4). One point was attributed for each correct answer. In the graduated number line task children placed a fraction (4 items) or the number 1 (4 items) on a graduated number line on which 0 and another fraction were indicated (e.g. Place 2/9 on the graduated number line, knowing 0 and 5/9). One point was given if they placed the fraction at the right graduation. Arithmetic operations included 8 additions and subtractions with same or different denominators, 4 multiplications of fractions and 4 multiplications of a fraction by an integer. For the simplification task, pupils were asked to reduce 4 fractions in lowest terms. 30 minutes were allocated for children to complete the paper and pencil tests. 

#### Comparison task

90 pairs of fractions were used in this task (stimuli are shown in Table S1). Denominators could be between 2 and 9, and numerators between 1 and 8. The global magnitude of fractions was always smaller than 1. Stimuli were grouped into three Global distance conditions: Close (global magnitude difference < 0.3), Medium (0.3 < global magnitude difference < 0.5) and Far (global magnitude difference > 0.5). Pairs of fractions were equally distributed along the three conditions.

There were five different categories of stimuli: (A) 18 pairs of fractions with the same numerator (Mean global distance = 0.30), (B) 18 pairs of fractions with the same denominator (Mean global distance = 0.31), (C) 6 pairs of fractions for which the numerator of fraction 1 and the denominator of fraction 2 were the same (Mean global distance = 0.31), (D) 6 pairs of fractions for which the denominator of fraction 1 and the numerator of fraction 2 were the same (Mean global distance = 0.30), and (E) 42 pairs of fractions with no common components (Mean global distance = 0.30). The size of the stimuli was 250 pixels x 250 pixels. Stimuli were randomly presented. 

In order to control for interrelationships between numerators, denominators and global distance we ran correlation analyses between global and component distances of fraction pairs. There was a significant positive correlation between global distance and distance between numerators (r = 0.476; *p*<0.001), and a marginally significant correlation between global distance and distance between denominators (r = -0.183; *p* = 0.084). There was no significant correlation between distance between numerators and distance between denominators (r = 0.044; *p* = 0.0683).

#### Matching task

135 pairs of fractions were distributed among three conditions: Identical, Equivalent and Different. There were 15 pairs of Identical fractions (e.g. 1/2_1/2), 30 pairs of Equivalent fractions (e.g. 1/2_2/4) and 90 pairs of Different fractions (e.g. 1/2_2/3). The 90 stimuli of Different fractions were the same as the pairs used in the Comparison task. 

### Procedure

Children first carried out the paper and pencil test as a group and then carried out the experimental tasks individually. There were two experimental tasks: a comparison task and a matching task. In both tasks, stimuli were presented on the screen of a computer using the Presentation program (Neurobehavioral systems). Black characters were presented on a white background. In each trial a fixation cross appeared on the screen for 300 msec, followed by a 200 msec blank screen. The pair of fractions stayed on the screen for 7000 msec or until the participant gave a response, followed by a 200 msec inter-stimuli interval. Two different types of vinculums (horizontal and diagonal) and two different types of fonts (arial and brush) were used. Each pair of fractions was made of different vinculum and different fonts to change the physical appearance of each fraction. The variation of fonts and vinculums was introduced to get participants’ attention focused on the semantic content of the stimuli rather than on their physical aspect [33]. The order of stimulus presentation was randomized. 

In the Comparison task, participants were asked to decide which of two fractions was larger. If the larger fraction appeared on the left of the screen, they had to press “q” key, if it appeared on the right of the screen, they had to press “m” key on an AZERTY keyboard. In the Matching task, they had to decide whether pairs of fractions were same or different. There were three conditions: Identical (e.g. 1/2_1/2), Equivalent (e.g. 1/2_2/4) and Different (e.g. 1/2_2/3). Pairs of fractions were considered to be the same when they represented the same quantity (i.e. Identical and Equivalent conditions). Participants had to press “m” key when fractions were the same. In the Different condition, fractions did not represent the same quantity. When pairs of fractions were different, participants had to press “q” key. Participants were allowed to take a short break between the tasks. Participants were explicitly told to ignore the physical appearance of the stimuli. The order of the computer-based tasks was counterbalanced. The duration of the computer-based tasks was approximately 25 minutes. 

### Data analysis

Trials on which there was an incorrect response were removed prior to reaction times (RT) analyses. Trials in which RT was greater than the participant's mean RT plus three standard deviations were excluded from analyses (2.8% of the trials in the matching task, and 1.9% of the trials in the comparison task).

First of all, a MANOVA was run on accuracy in all of the paper and pencil subtests (Estimation, Comparison, Graduated Number Lines, Arithmetic Operations and Simplification) with Grade (3 levels: Grade 5, Grade 6, Grade 7) as a fixed factor. Tukey-HSD tests were used for post-hoc comparisons.

Second, ANOVAs were run in order to keep comparability with the whole literature. The main objective was to detect whether there was a representation of global fraction magnitude. Hence, various analyses focused on the global distance effect. First, accuracy (percentage of correct responses) and mean RT (milliseconds) was determined in each condition in each participant. The effect of global numerical distance was directly compared across tasks by analyzing the stimuli from the Different Condition of the matching task and stimuli from the Comparison task. Those stimuli were identical in both tasks. Three categories of stimuli were defined according to the global distance between pairs of fractions: close, medium, and far distance. A repeated measures ANOVA was used on accuracy and RT. There was one between-subjects factor, Grade (grade 5, grade 6, grade 7 and adults); and two within-subjects factors, Task (Matching and Comparison) and Global Numerical Distance (close, medium and far). Pairwise comparisons in the above and all other analyses were done by post-hoc Tukey-HSD tests. In order to control for the development of speed of processing, proportionally transformed RT were also analyzed [34]. Proportionally transformed RT was computed for two different magnitudes of numerical distance: Distance Level 1: (close distance – medium distance) / far distance; Level 2: (close distance – far distance) / far distance. These values were entered into a Grade × Task × Distance Level ANOVA. It is to note that we always divided by the value of far distance values in order to assure that all values are in a common metric. 

Third, in order to further specify distance effects we computed the mean slope of the distance effect in each task for both accuracy and RT by taking the mean of close minus medium and medium minus far distance values for original RT and ran a Grade × Task ANOVA on slope values. Moreover, in order to see whether the slope was significantly different from zero, the value of the slope in in each Task and Task × Grade cell was tested against zero by running one sample two-tailed t-tests [35]. 

A similar analysis was done on proportionally transformed RT data. In proportionally transformed data the slope of the distance effect was computed by calculating two levels of distance and averaging them: Level 1: (close distance – medium distance) / far distance; Level 2: (medium distance – far distance) / far distance. These values were also analyzed by a Grade × Task ANOVA. We tested for the presence of speed/accuracy trade-offs by correlating accuracy and RT scores in the whole sample and in each group separately at an alpha level of *p* = 0.05. The above Grade × Task ANOVAs on slope values were also run when taking the slope of the accuracy global distance effect as a covariate. 

Fourth, stepwise multiple regression analyses investigated whether global and/or componential numerical distance values predicted mean RT in each task. These were in the same way as in Ischebeck et al. [25]. In the first stage of the analysis, the independent variable which best correlated with the dependent variable (RT) was included in the model. In the second stage, the next independent variable with the highest partial correlation with the dependent variable was also included in the model. The process is repeated until the addition of the remaining independent variable does not significantly increase adjusted R^2^ or until the last variable is included. The entrance criterion was set at *p* ≤ 0.05 and the exit criterion at *p* ≤ 0.1. The final model is reported for each analysis. In the comparison task the regression analyses were also run in each individual and the proportion of participants demonstrating significant global distance regression outcome was investigated by examining binomial distribution probabilities. In addition, the individual beta values were used to represent the slope of the global distance effect at the individual level. In order to test whether this slope differed by grade, the beta values from the Comparison task (there were no significant global distance regression results in the other task) were entered into an ANOVA with a Grade factor.

Fifth, in order to keep consistency with the data analysis of Meert et al. [8], a linear mixed model analysis was run on RT with grade (grade 5, grade 6, grade 7 and adults), global distance, distance between numerators, distance between denominators, type of fraction ((A) same numerators, (B) same denominators, (C) numerator of fraction 1 and denominator of fraction 2 are the same, (D) denominator of fraction 1 and numerator of fraction 2 are the same, and (E) no common components) and task (comparison vs. matching) as fixed main effects [8]. The model also included the following interactions: global distance x grade, global distance x task and global distance x type of pair x task. Two random factors were also included in the model: the random intercept for participants and the random intercept for the pairs of fractions. 

Finally, we computed correlations between the slope values of the distance effect in each task for both accuracy and RT with performance on the paper and pencil tests. 

## Results

### Paper and pencil tests

Table **1** shows results in paper and pencil tests. A MANOVA was run on accuracy for the 5 subtests of the paper and pencil tasks with Grade as a fixed factor. The MANOVA showed a significant Grade effect, *F*(2, 35) = 8.65, η^2^_p_ = 0.19, *p* = 0.001. Post hoc test showed that grade 5 children had worse performance than grade 6 and grade 7 children (*p* < 0.05). There was a grade effect in the comparison task; post-hoc test showed that grade 5 children performed worse than grade 6 and grade 7 children (*p* < 0.001). There were significant grade differences in the arithmetic operation; post-hoc test showed that grade 5 children performed worse than grade 6 and grade 7 children (*p* < 0.05). Post hoc test also showed significant differences between grades in the graduated number line subtest: Grade 5 children performed worse than grade 6 and grade 7 children (*p* < 0.001). Finally, post hoc test also showed significant grade differences in the simplification subtest: Grade 5 children performed worse than grade 6 and grade 7 children (*p* < 0.05).

**Table 1:** Mean percentage and standard deviations of correct responses for the different questions of the paper and pencil test.

	**Estimation**	**Comparison**	**Number Lines**	**Operations**	**Simplification**
**Grade 5**	66.2 ± 8	68.4 ± 4	28.6 ± 2	19.1 ± 8	35 ± 3
**Grade 6**	90.2 ± 5	87.2 ± 5	63.7 ± 8	55.9 ± 9	66.3 ± 5
**Grade 7**	92.4 ± 5	93.5 ± 5	82.8 ± 7	63.3 ± 9	88.2 ± 5

### Experimental tasks

#### ANOVAs on original data

**Figure 2:**
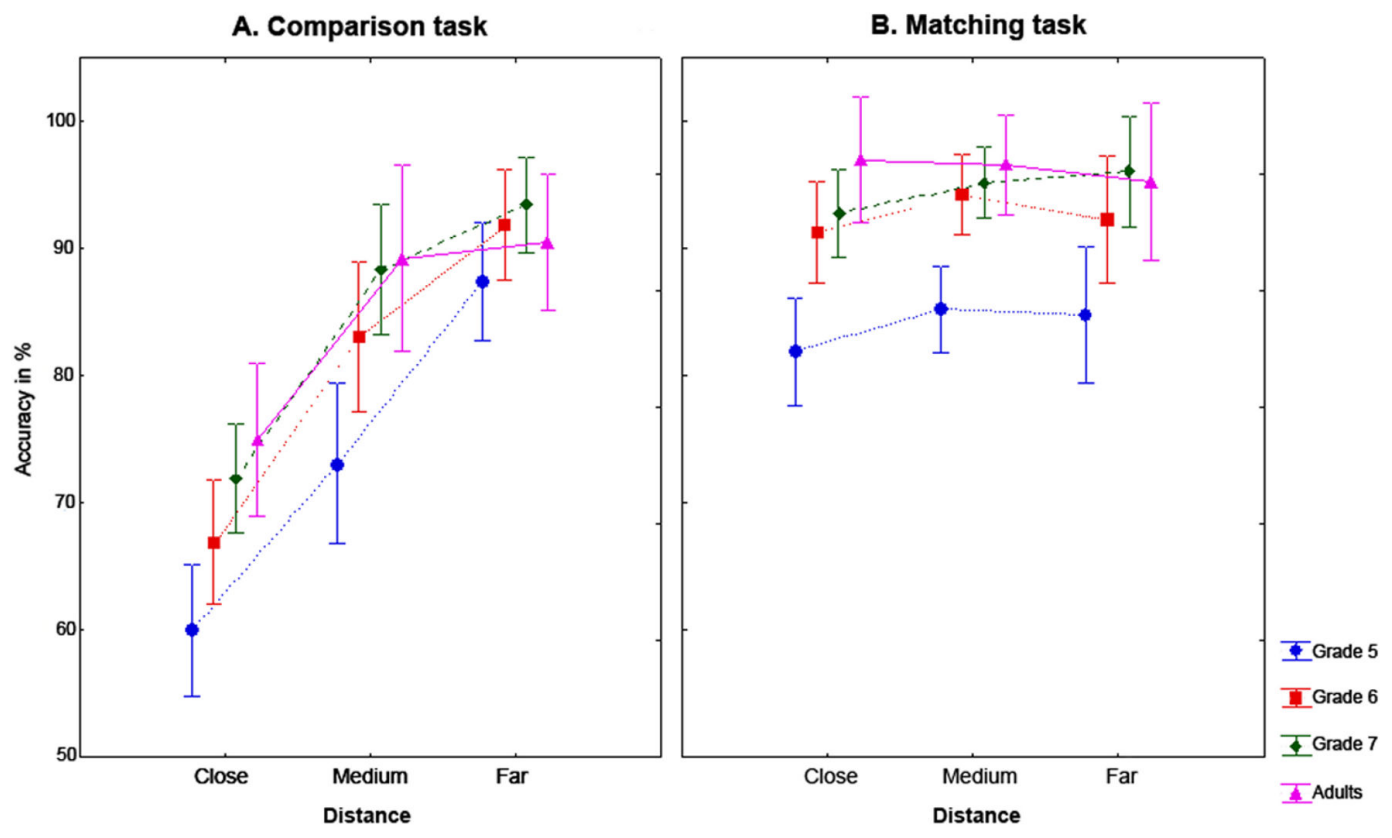
Accuracy for both tasks. This figure shows the accuracy for the Comparison task (A) and Matching task (B) by global distance (close, medium and far) for each grade. Vertical bars denote 95% confidence intervals.

Accuracy in both tasks is shown in Figure **2**. A Grade × Task × Numerical distance ANOVA was run on data collected in the different condition of both tasks. There was a main effect of grade, *F*(2,70) = 16.32, η^2^_p_ = 0.32, *p* < 0.001, as accuracy increased with grade (Mean and standard deviation: Grade 5 = 78% ± 16; Grade 6 = 84% ± 13; Grade 7 = 87% ± 11.3; Adults = 87%.3 ± 7.2). Post-hoc tests showed that grade 5 children performed worse than grade 6 children, grade 7 children and adults (all *ps* < 0.001). There was a main effect of task, *F*(2,64) = 53.20, η^2^_p_ = 0.43, *p* < 0.001, as overall there were more correct responses in the matching task (93%) than in the comparison task (84%). There was a significant Task x Distance interaction, *F*(2,64) = 77.93, η^2^_p_ = 0.53, *p* < 0.001. Post hoc tests showed that there was an expected graded distance effect in the Comparison task as all levels of distance were significantly different from each other (all *ps* < 0.001). The levels of numerical distance were not different from each other in the matching task. 

**Figure 3:**
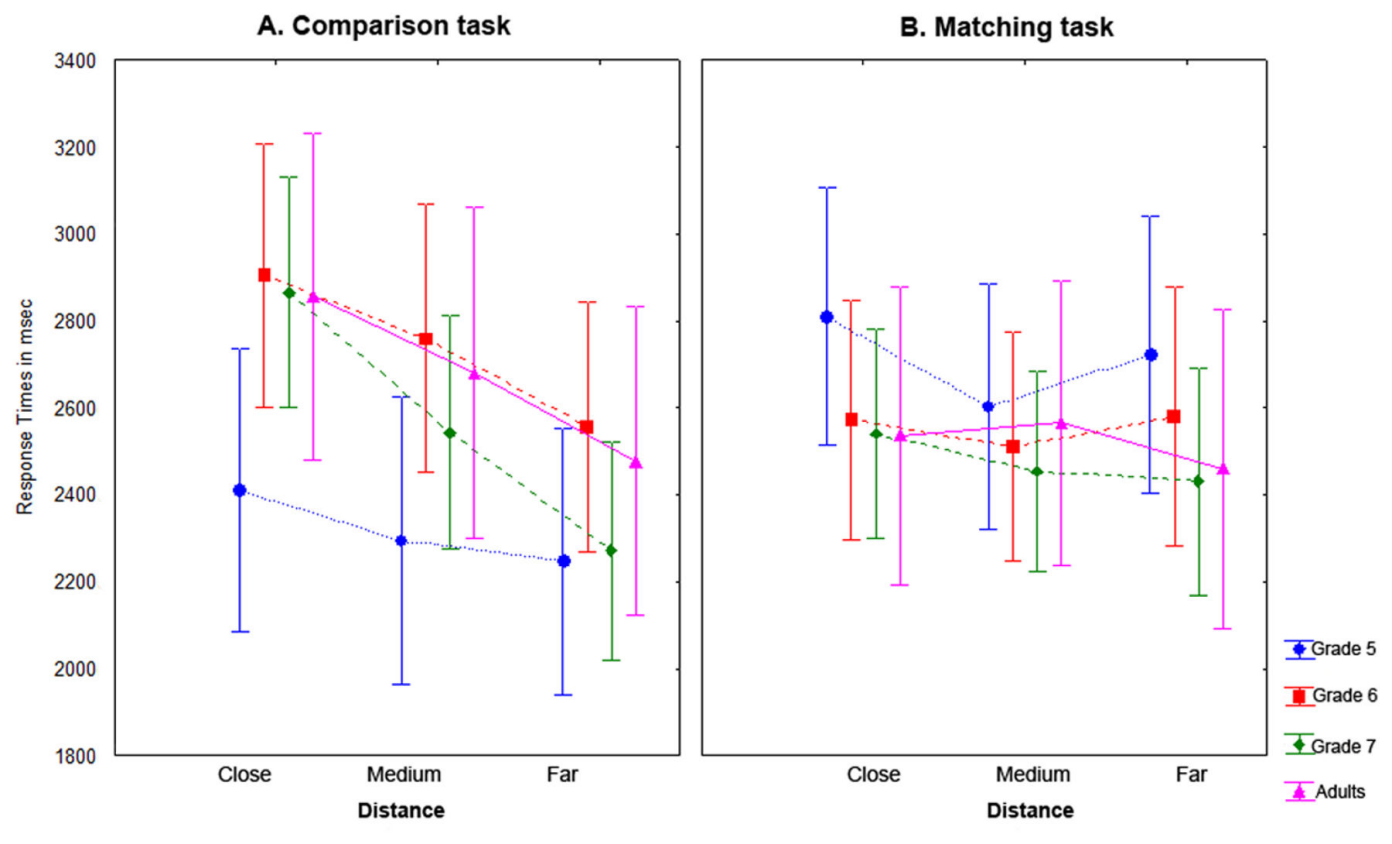
RT for both tasks. This figure shows RT for the Comparison task (A) and Matching task (B) by global distance (close, medium and far) for each grade. Vertical bars denote 95% confidence intervals.

RT in both tasks are shown in Figure **3**. A Grade × Task × Numerical distance ANOVA showed a significant task effect, *F*(1, 168) = 11.24, η^2^_p_ = 0.22, *p* = 0.002. Overall, participants were faster in the Matching task (M = 2514 ± 486) than in the Comparison task (M = 2776 ± 641). Results also showed a significant Task × Distance interaction, *F*(56, 168) = 2.12, η^2^_p_ = 0.75, *p* = 0.007. Post hoc Tukey tests showed that there was an expected graded distance effect in the Comparison task (all levels of distance were significantly different from each other: all *ps* < 0.001). There was no graded distance effect in the matching task. There was a Task × Grade, *F*(3, 168) = 24.97, η^2^_p_ = 0.65, *p* < 0.001, interaction as the comparison task was responded slower than the matching task in grade 6 (comparison – matching RT = 186 ms), grade 7 (comparison – matching RT = 86 ms) and adults (comparison – matching RT = 151 ms) but the pattern of results was the opposite in grade 5 (comparison – matching RT = -394 ms). Further, there was a Task × Distance × Grade interaction, *F*(2, 132) = 3.21, η^2^_p_ = 0.08, *p* = 0.015. Grade × Task × Distance post-hoc contrasts showed that in grade 5 there was no difference between levels of distance (all *ps* > 0.56). In grade 6 the difference between close and far distance was significant (*p* < 0.001), the difference between medium and far distance was marginal (*p* = 0.09), and the difference between close and medium distance was not significant (*p* > 0.10) in the Comparison task. In grade 7 and adults all levels of distance were significantly different from each other (all *ps* < 0.001) in the Comparison task.

Another ANOVA examined proportionally transformed RT data and delivered perfectly consistent results with the ANOVAs run on raw RT.

#### The slope of the distance effect in original data

**Figure 4:**
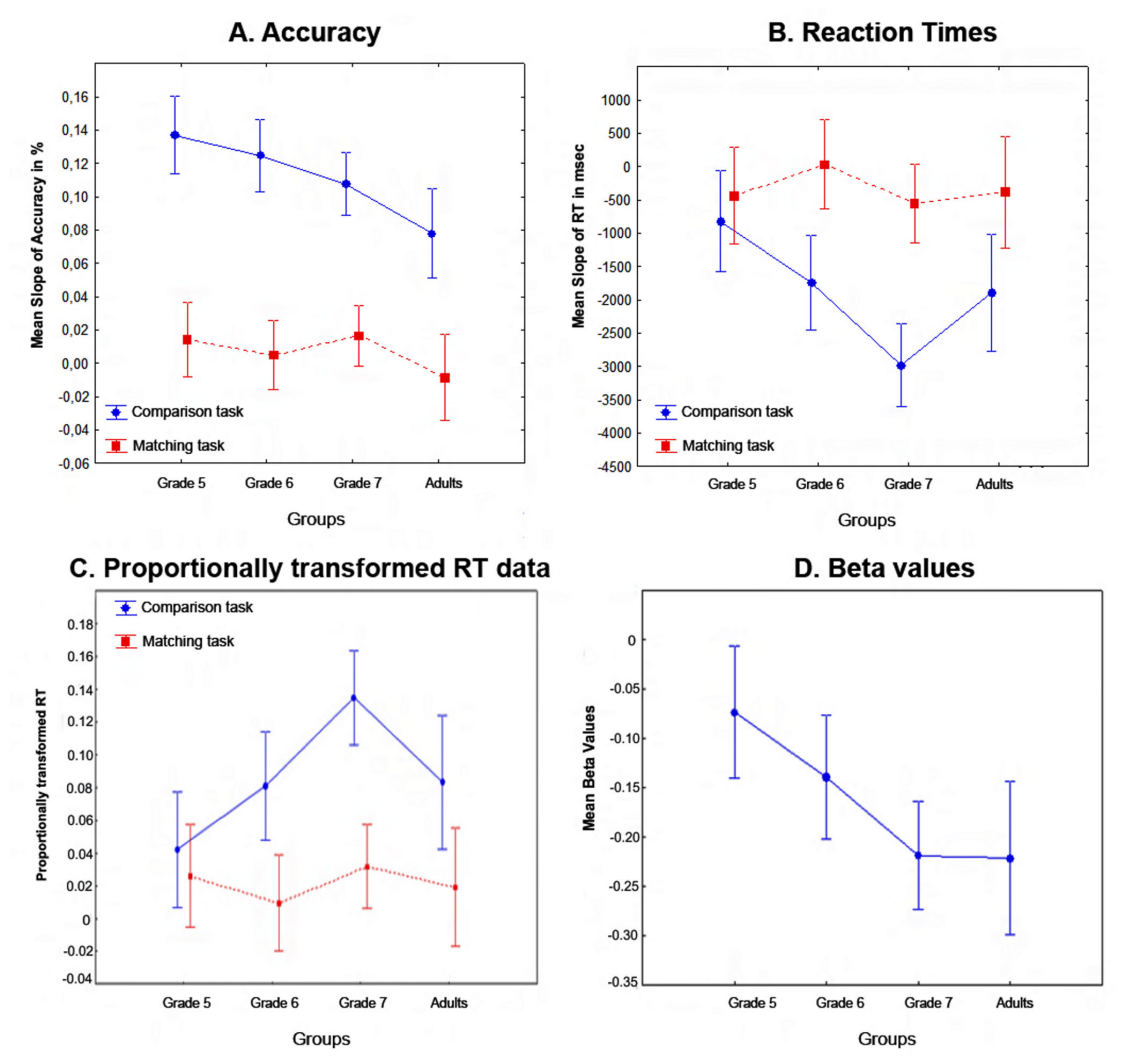
Mean slopes. This figure shows mean slopes of accuracy (A) and RT distance effects (B); Average slope of the distance effect for proportionally transformed RT (C); and mean beta values for each group (D).

The mean slopes of accuracy distance effects are shown in Figure **4A**. A Grade × Task ANOVA on accuracy slope values showed only a Task main effect, *F*(1, 70) = 155.75, η^2^_p_ = 0.44, *p* < 0.001 reflecting that the slope of the distance effect was steeper in the comparison (0.123%) than in the matching task (0.011%). The mean slopes of RT distance effects in original RT are shown in Figure **4B**. There was a Grade main effect, *F*(1, 70) = 5.19, η^2^_p_ = 0.38, *p* = 0.008. There was also Task main effect, *F*(1, 70) = 33.53, η^2^_p_ = 0.19, *p* < 0.001, and a Grade × Task interaction, *F*(2, 70) = 5.12, η^2^_p_ = 0.25, *p* = 0.008. As all analysis outcomes were similar to the outcome of proportional RT analysis, we only describe post-hoc results for the proportional RT analysis below. 

One sample t-tests against zero checked whether the slope of accuracy and RT distance effects were significantly different from zero. An analysis run an all groups’ data showed that the slope of both the accuracy, *t*(87) = 19.29; *p* < 0.001, and RT, *t*(87) = -8.71; *p* < 0.001. Distance effect differed from zero in the comparison task but not in the matching task. There were similar results in grade 5, accuracy: *t*(19) = 11.29; *p* < 0.001; RT: *t*(19) = -2.1; *p* = 0.048, grade 6 accuracy: *t*(22) = 9.46; *p* < 0.001; RT: *t*(22) = -4.81; *p* < 0.001, grade 7 accuracy: *t*(29)= 13.87; *p* < 0.001; RT: *t*(29) = -9.03; *p* < 0.001, and adults accuracy: *t*(14) = 6.50; *p* < 0.001; RT: *t*(14) = -5.33; *p* < 0.001. In addition, the slope of the accuracy distance effect in the matching task also became significantly different from zero in grade 7, t(29) = 2.49; *p* = 0.018. 

#### The slope of the distance effect in proportionally transformed RT data

Slopes determined by proportionally transformed RT data are shown in Figure **4C**. According to the Grade × Task ANOVA there was a Grade main effect, *F*(3,84)=3.54, η^2^_p_ = 0.11, *p* = 0.018, because the slope was larger in grade 7 than in any other grade (*p* = 0.003 for all). There was a Task effect, *F*(1,84)=35.37, η^2^_p_ = 0.30, *p* < 0.0001, and a Task × Grade interaction, *F*(3,84) = 3.2, η^2^_p_ = 0.10, *p* = 0.027. The Task × Grade interaction was present because the slope was steeper in the Comparison task than in the Matching task in grade 6 (*p* < 0.001), grade 7 (*p* < 0.0001) and in adults (*p* = 0.01) but not in grade 5 (*p* = 0.5).

At the level of the whole sample only the large global distance condition showed speed/accuracy trade-off, *r* = -.27; *p* = 0.008. Separate analyses on data of age groups showed that this effect was driven exclusively by Grade 7 children, *r* = -0.48; *p* =0.007. All other correlations remained non-significant (lowest *p* = 0.17).

In order to account for potential speed/accuracy trade-offs we ran the analysis of RT slope data from the comparison task with a Grade factor taking the slope of accuracy data as a covariate. The Grade effect remained highly significant both when considering original RT, *F*(3,83)=6.29; η^2^_p_ = 0.18; *p* < 0.001, and proportionally transformed RT, *F*(3,83)=3.21; η^2^_p_ = 0.19; *p* = 0.027).

#### Regression analyses

Stepwise multiple regression analyses assessed whether global and/or componential distance effects predicted RT in each age group. In the matching task there were only componential distance effects (see Table **2**). The regressions yielded a total *R* = 0.42 (adjusted *R* ^2^ = 0.16) in grade 5, *R* = 0.41 (adjusted *R* ^2^ = 0.17) in grade 6, *R* = 0.32 (adjusted *R* ^2^ = 0.09) in grade 7, and *R* = 0.18 (adjusted *R* ^2^ = 0.03) in adults. The global distance effect was not a significant predictor of RT. 

**Table 2:** Results of the stepwise multiple regression analyses by group.

	**Coeff.**	**Comparison task**			**Matching task**		
**Group**		**Num**	**Denom**	**Global**	**Num**	**Denom**	**Global**
**Grade 5**	**β**	-0.40	-0.01	0.03	-0.17	0.36	0.02
	***t* (86)**	-3.06	-0.37	0.79	-2.1	4.77	0.68
	**P. Corr.**	-0.40**	-0.01	0.02	-0.18*	0.37**	0.02
	**Z. Corr.**	-0.16**	-0.01	-0.03	-0.02	0.02	-0.02
**Grade 6**	**β**	0.03	0.04	-0.38	-0.18	0.35	-0.03
	***t* (86)**	0.91	1.43	-2.80	-2.24	4.32	-0.79
	**P. Corr.**	0.02	0.04	-0.37*	-0.19*	0.35**	-0.02
	**Z. Corr.**	-0.06**	0.08**	-0.12**	0.01	0.05**	-0.02
**Grade 7**	**β**	-0.29	0.04	-0.68	-0.04	0.32	-0.02
	***t* (86)**	-2.25	1.80	-5.70	-0.06	3.84	-0.05
	**P. Corr.**	-0.28*	0.04	-0.56**	-0.04	0.32**	-0.02
	**Z. Corr.**	-0.07**	0.11**	-0.20**	0.01	0.07**	-0.04
**Adults**	**Β**	-0.20	0.09	-0.64	-0.02	0.12	-0.002
	***t* (86)**	-1.86	1.09	-7.78	-0.525	7.34	-0.05
	**P. Corr.**	-0.19**	0.09*	-0.64**	-0.02	0.12**	-0.002
	**Z. Corr.**	-0.15**	0.1**	-0.18**	-0.03	0.06**	-0.03

Coeff. = Coefficients; P.Corr = Partial Correlations; Z. Corr = Zero-Order Correlations; Num = Distance between numerators; Denom = Distance between denominators; Global = Global Distance. * *p* ≤ 0.05. ** *p* ≤ 0.01.

In the Comparison task, the regressions yielded a total *R* = 0.40 (adjusted *R* ^2^ = 0.16) in grade 5, *R* = 0.39 (adjusted *R* ^2^ = 0.21) in grade 6, *R* = 0.58 (adjusted *R* ^2^ = 0.31) in grade 7, and R = 0.40 (adjusted R^2^ = 0.30) in adults. Global distance predicted RT in Grade 6, Grade 7, and adults (see Table **2**). The relationship appeared because participants were faster as the global distance between fractions got larger. In addition, the distance between numerators predicted RT in Grade 5, Grade 7, and adults. Distance between denominators predicted RT in adults. 

Mean beta values for significant global distance as a predictor of RT are shown in Figure **4D**. An ANOVA with a Grade factor compared the global distance regression beta values across participant groups. The Grade effect was significant, *F*(3,84)=4.63, η^2^_p_ = 0.14, *p* = 0.005. Post-hoc tests showed that beta was more negative in grade 7 (*p* = 0.007) and in adults (*p* = 0.0266) than in grade 5. Correlations between beta values and performance in paper and pencil tasks are shown in Table 3.

**Table 3:** Correlations between task performance and accuracy and RT distance effect slopes.

	**PPEst**	**PPComp**	**PPOp**	**PPNbLine**	**PPSimp**	**CompAcSlope**	**CompRTSlope**	**MAtchAcSlope**
**PPComp**	0.56**							
**PPOp**	0.46**	0.54**						
**PPNbLine**	0.51**	0.66**	0.70**					
**PPSimp**	0.38**	0.50**	0.56**	0.70**				
**CompAcSlope**	-0.29*	0.43**	-0.28*	0.43**	-0.26*			
**CompRTSlope**	-0.18	-0.34**	-0.31**	-0.44**	-0.29**	0.15		
**MatchAcSlope**	0.07	0.15	0.01	-.02	0.04	-.01	-0.25	
**MatchRTSlope**	-0.02	-0.16	0.05	0.05	-0.19	0.13	0.19	0.05

Abbreviations: PP = Paper and pencil; Est = Estimation; Comp = Comparison; Op = Operations; NbLine = Number Line; Simp = Simplification; Ac = Accuracy; RT = Response Times.* *p* ≤ 0.05. ** *p* ≤ 0.01.

#### Linear mixed model analysis

In order to further confirm results, a linear mixed model was run on RT. Results were coherent with the ones obtained with the ANOVAs. There was a significant effect of Grade (*p* =0.001), a significant Global Distance effect (*p* = 0.001), a significant Grade x Global Distance interaction (*p* = 0.046), a significant Global Distance x Task Interaction (*p* < 0.001) and a significant Global Distance x Grade x Task interaction (*p* = 0.021). 

#### Correlations between distance effects and paper and pencil task performance

Table **3** shows correlations between task performance, accuracy, original and proportionally transformed RT distance effect slopes as well as beta values. Performance on each of the paper and pencil tasks positively correlated with performance on any other paper and pencil tasks. There were negative correlations between paper and pencil tasks and the accuracy and RT distance effect slope in the comparison task. However, the negative correlation between the estimation task and the distance effect slope in the comparison task did not reach significance. There were no correlations between tasks and the accuracy and RT distance effect slope in the matching task. This is not surprising as no global distance effect was found in the matching task. 

#### Individual level analyses

In order to assess individual differences, the presence of the global distance effect was tested by running regression analyses on individual datasets in the Comparison task. In grade 5 three out of 20 (15% of grade 5 children; binomial cumulative probability of number of participants ≥ 3: *p* = 0.99), in grade 6 eight out of 23 (34.8% of grade 6 children; binomial cumulative probability of number of participants ≥ 6: *p* = 0.89), in grade 7 nineteen out of 30 (63.3% of grade 7 children; binomial cumulative probability of number of participants ≥ 19: *p* = 0.0494) pupils showed a significant global distance effect, and in adults ten out of 15 participants showed a significant global distance effect (66.6% of adult participants; binomial cumulative probability of number of participants ≥ 10: *p* = 0.0593). A Kruskal-Wallis test was run to compare the number of participants showing a significant global distance effect across grades. Result showed a significant effect between grades, χ^2^ = 12.7, *p* = 0.005; Mean ranks: grade 5 = 34; grade 6 = 38; grade 7 = 50; adults = 56. The Mann-Whitney U test was used as a post hoc test for difference between two grades and showed significant differences between grade 5 and grade 7 (*p* = 0.012) and between grade 5 and adults (*p* = 0.009). There were also significant differences between grade 6 and grade 7 (*p* = 0.04) and between grade 6 and adults (*p* = 0.015). 

## Discussion

Fractions are crucial in mathematics learning. They are fundamental to understand higher-level mathematics topics such as algebra. However, fractions pose a serious learning problem to young children which seems to contrast with the proposal of an evolutionarily based global magnitude representation. Here, we examined whether a global magnitude representation of fractions is automatically activated, in two conceptually different magnitude analysis tasks. We used exactly the same pairs of fractions from both tasks to answer our questions. Second, we examined changes during child development revealed by modulations in the slope of the global distance effect. Third, we not only studied group level effects but also examined the presence of a global fraction representation in each individual. Fourth, we examined the functional significance of the global magnitude representation of fractions by relating it to mathematical performance on paper and pencil tests assessing children's knowledge about fractions. Fifth, we compared the outcome of several methods targeted at detecting the development of the global magnitude representation of fractions.

### The activation of global fraction magnitude representation is not automatic for all magnitude-related tasks and depends on task demands

We used two magnitude comparison tasks which according to previous results are both robust measures of number processing skills [27] and tap into conceptually different aspects of global magnitude activation [29]. The same stimuli were used in both tasks. Results were clear cut. ANOVAs demonstrated robust Task × Global distance interactions: There were strong global distance effects in the comparison task in grades 6, 7 and adults whereas there were no global distance effects in the matching task. Results were confirmed by multiple testing corrected pair-wise comparisons of different levels of global distance. Additional regression analyses controlling for the effects of numerator, denominator and global distance effects in turns, found that global distance was the strongest predictor in the comparison task in grades 6, 7 and adults. In contrast, only componential (numerator and denominator) distance effects predicted RT in the matching task. This indicates that the matching task was solved without accessing the global magnitudes of fractions. 

The fact of finding a global distance effect in at least one of the tasks suggests that the global magnitude representation of fractions can get activated under certain conditions. This is a necessary precondition of any further associations between global magnitude and response options. As outlined in the introduction this is not a trivial finding because relative magnitude relations between the to-be-compared fractions are not as evident as between integers. However, the dissociation between the comparison and matching tasks suggests that the activation of the global magnitude representation of fractions depends on task context/demands and is not automatic in all tasks involving explicit magnitude judgements, i.e. global fraction magnitude is not necessarily accessed in all fraction tasks. We suggest that the global distance effect appears only in tasks where the explicit labelling of smaller/larger relations is requested (like in the comparison task). However, according to Van Opstal and Verguts [29] such tasks are confounded by stimulus-response associations and cannot provide a pure measure of the magnitude representation. Previous studies did not consider the above possibility.

Finding only componential distance effects in the matching task is compatible with conclusions drawn by Bonato et al. [4] and Kallai and Tzelgov [5]. However, some of these previous findings may also have been due to task context. For example, Meert et al. [7] and Schneider & Siegler [24] have demonstrated that the activation of the magnitude of fractions depends on the type of fractions used. Bonato et al. [4] used only fractions of the form 1/x in their Experiments 1 and 2. Obviously, this type of fractions encourages participants to focus solely on the denominators. Further, in their experiments 3 and 4, the size of the numerators was always consistent with the global size of the fractions, again, allowing a componential strategy to be successful. Kallai and Tzelgov [5] contrasted numerical and physical comparison of fractions with a numerical Stroop paradigm. Results showed that participants preferred to use strategies based on integer numbers, indicating that there was no unique representation of the global magnitude of fractions in long-term memory. However, again, in their first and fourth experiment, only fractions of the form 1/x were used. Therefore, participants could base their judgement on the denominators only. In the second experiment, proper and improper fractions were used, but the larger the numerator was, the larger the global magnitude of the fraction was, encouraging the use of componential strategies. In the third experiment, familiar fractions were used and they observed hybrid strategies in the numerical comparison. This indicates that the nature of the fractions used could have influenced the type of processing. 

It is important to point out that it is unlikely that global distance effects in our study were confounded by denominator and numerator distance effects. First, we used partial correlations, successively controlling for numerator, denominator and global distance effects. Second, the pattern of results also makes any confounds unlikely. This is because, the correlation of numerator and denominator distance with RT was either non-significant or had exactly the opposite sign and the absolute value of both correlations was smaller than the correlation of global distance with RT and also had opposite sign correlations with global distance (e.g. in adults: global distance correlation in the comparison task, r = -0.635; numerator r = -0.194; denominator r = +0.085). Therefore, the global distance effect cannot be explained by the joint effect of numerator and denominator distance, rather it can be considered a strong effect on its own.

### Age-related changes in accessing the global magnitude representation

Overall accuracy was lower in grade 5 (78%) than in grades 6, 7 and adults (84-87%), the later three groups not being significantly different from each other. Moreover, RT did not differ in grades 6, 7 and adults. This suggests that competence with the kinds of fractions used here reaches close to adult levels in grades 6 and 7. Regressions analyses demonstrated that global distance became a significant predictor of RT in the comparison task from grade 6 upwards. ANOVA results and stringent Group × Task × Global Distance post-hoc contrasts were consistent with regression results, demonstrating global distance effects between all levels of distance in grade 7 and adults. Not all levels of global distance were significantly different from each other yet in grade 6 and there were no significant differences between levels of global distance at all in grade 5 pupils. The slope of the global distance effect was significantly larger in the comparison task than in the matching task in grades 6 and 7 and adults but not in grade 5. 

We not only examined group level data but also statistically evaluated the significance of regression results in each individual. The proportion of participants demonstrating global distance effects increased with development and was 15%, 35%, 63% and 67% in grades 5, 6, 7 and adults, respectively. The number of participants showing the global magnitude representation signature became significantly different from chance in grade 7 and in adults. Statistical comparison of the number of participants with global distance effects across groups also showed that the number of participants with global distance effects increased between grades 5 vs. 7, 6 vs. 7, 6 vs. adults but was about the same between grades 5 vs. 6 and 7 vs. adults. The above group and individual-level data both suggest continuous developmental progression in the use of global fraction magnitude information in the comparison task.

As discussed, the fact of detecting global distance effects at least suggests that the global magnitude representation of fractions is exploited in a certain task context. Hence, first of all, these findings suggest that grade 5 children do not yet rely on global fraction magnitude even if the task context facilitates it. Our findings suggest that pupils progressively have better access to the global magnitude representation of fractions during grades 5, 6 and 7 in a magnitude comparison task. Our results are compatible with the findings of Siegler, Thompson and Schneider [36] who also showed that fraction knowledge is acquired at older ages than whole number knowledge. They argue that knowledge about the magnitude of fractions is less accurate than whole number knowledge at least until grade 8 [36]. It is interesting to note that in accuracy global distance effects were significant already at grade 5. It is an interesting avenue for further research to explore this accuracy vs. RT dissociation in early stages of fraction acquisition.

The slope of the global distance effect was the largest in grade 7 children, even larger than in adults. First, this suggests that the slope increased from younger to older children. This finding differs from the results of Meert et al. [8] who did not show a change in the slope of the distance effect in grade 5 and 7 children. As the studies involved children of the same nationality from the same school system at the same age this discrepancy may be attributed to the potentially higher power of our study to detect changes in the distance effect slope. Second, our finding is in contrast to the classical findings of Sekuler and Mierkiewicz [21] who reported that the slope of the distance effect elicited by single-digit integer numbers decreased with age. We suggest that the slope increased in children because global magnitude became more readily accessible in older than in younger children. We suggest that our results are different from integer results [21,28] because the acquisition and mobilization of global fraction magnitude is much more demanding and happens at a later age than learning and activating the magnitude of integers. Several studies suggest that children very quickly acquire the meaning of integers during grade 1 [20,22]. In fact, most children probably learn some single Arabic digits already in kindergarten. Hence, after grade 1 a reduction in the distance effect slope may reflect more efficient resolution between levels of numerical distance [21]. In contrast, the initial learning of the global magnitude of fractions is a more effortful task. Hence, initially we could expect no distance effects with fractions (similarly, we could expect no numerical distance effects with single digits in children who do not know any digits, yet). Then, we could expect increasingly stronger distance effects as children gradually acquire a more global vs. component sense of fraction values, as observed here from grade 5 to 7. Even later, it could be expected that after learning to retrieve global magnitude values more efficiently, a reduction of the distance effect would be observed because of the more efficient resolution of categorical global distance differences. 

The above outlines potential age-related changes may have been observed in our study as RT data showed that the slope of the distance effect decreased from grade 7 to adults. Our data support the possibility that adults do not have better access (more automatic) to numerical magnitude of fractions. Such a decrease would be similar to the decrease of the integer distance effect as observed by Sekuler and Mierkiewicz [21]. The above suggest that during the whole developmental pathway a reversed U shaped curve could be expected. Initially distance effects were getting larger as global fraction magnitude information is becoming more accessible. Later, distance effects would get smaller (but would remain significant) because of either the more efficient resolution of global distance categories, or because adults have generally less practice with fractions than older children. Ultimately, this hypothesis could be tested by studies covering for example grades 5 to 12 of school. It is important to note that our data is also controversial because the analysis of beta values showed that the slope was the same in adults and in grade 7. This directs attention to the important fact that even slightly different measures can support theoretically very different conclusions. Hence, it is highly beneficial to present the outcomes of multiple analysis methods.

### Functional significance of global distance effects

Relationships between knowledge of fractions and global distance effect in the matching task and the comparison tasks were also investigated. Knowledge of fractions was assessed with three different tasks: estimation, comparison and graduated number lines, arithmetic operations and simplification [12,17,32]. Significant correlations were found between the global distance effect slope in the comparison task and knowledge of fraction. Accurate magnitude representations of fractions are crucial. In a recent study, we showed that children gained greater understanding of fractions after a training focusing on their magnitude [37]. Our findings are also in line with the results of Siegler et al. [36] who found strong correlations between fraction arithmetic and the understanding of the magnitude of fractions in grade 6 and grade 8 children. The estimation task only correlated with the global distance effect slope on accuracy and not on RT. 

Our results are also compatible with the findings of De Smedt, Verschaffel and Ghesquière [38] who also showed that performance in a numerical comparison task involving natural numbers in grade 1 is predictive of individual differences mathematics achievement in grade 2. In a recent study, a negative correlation between symbolic distance effect and arithmetic skills was also found in 8-to-10-year-old children [39]. We have not measured IQ. Therefore, it remains a question whether global distance and performance correlations reflect general IQ effects. However, Mazzocco and Devlin found that Grade 6 children with a mean IQ of 116 only gave 59% correct responses when asked to order a set of fractions [40]. That is, high IQ may not guarantee good performance in fraction tasks.

It is important to point out some limitations of our study. First, we collected cross-sectional data and may have biased data towards high performers by selecting children who achieved at least 60% correct performance on fractions tasks. However, this selection seemed necessary so that we could avoid including children who guessed solutions. Second, it is currently difficult to decide (and it is beyond the scope of this paper) exactly what strategy was used for processing identical and equivalent fractions in the matching task.

### Conclusion

 Global distance effects were observed only in the comparison task but not in the matching task. Our data suggest that the global magnitude representation of fractions can be accessed in certain task context. However, global magnitude representation is not automatic in all tasks involving magnitude judgements. Global distance effects in the comparison task were probably confounded by general psychophysical comparison processes and/or stimulus-response mappings. Global distance effects appeared in RT from grade 6 onwards. This suggests that the task-development activation of the global magnitude representation undergoes development. The slope of the global distance effect increased during early fraction learning and declined or stayed steady by adulthood. This demonstrates that the development of the fraction global distance effect differs from that of the integer distance effect. Individual data reflected group level findings. Global distance effects from the comparison task showed correlations with mathematical performance on paper and pencil tests assessing children's knowledge about fractions. Even if global magnitude activation stems from an innate ability to process non-symbolic ratios, children still need to understand the relation between magnitudes conveyed by symbolic fractions. This learning process can be slow as children need to overcome the whole-number bias and learn a new symbolic system.

## Supporting Information

information S1 **Examples of questions used in the paper and pencil test.**
(DOCX)

Table S1 **Stimuli used in the experimental tasks.**
(DOCX)
