# Supplementary material for: The Development of the Mental Representations of the Magnitude of Fractions
Source: PLoS One. 2013 Nov 13;8(11):e80016. doi: 10.1371/journal.pone.0080016 (PMC3827422; doi:10.1371/journal.pone.0080016)
Supplement: information S1 — Examples of questions used in the paper and pencil test. (DOCX) [file pone.0080016.s001.docx]

Appendix S1: Examples of questions used in the paper and pencil test

**Estimation**

Without using a ruler, place
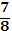

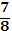
 on the line below


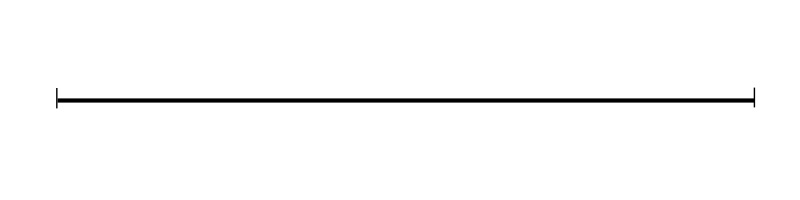


0 1

**Comparison**

Circle the larger fraction

1.
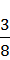

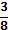

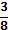

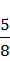

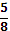

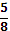

2.
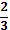

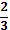

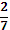

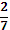

3.
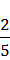

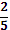

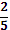

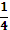

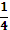


**Arithmetic operations**

Solve the following problems

a)
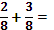
$\frac{2}{8}+\frac{3}{8}=$

b)
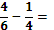
$\frac{4}{6}-\frac{1}{4}=$

c) $\frac{3}{4} x\frac{1}{6}=$

**Graduated number lines**

Place the number 1 on the graduated line below

1.
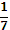

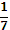


Place
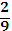

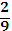
 on the graduated line below

1. $\frac{5}{9}$
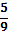


Place
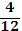

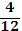
 on the graduated line below

0
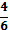

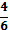


**Simplification**

Simplify the following fractions

1.
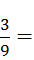

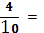
$\frac{4}{10}=$
2.
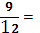
$\frac{9}{12}=$
