# Supplementary material for: The Development of the Mental Representations of the Magnitude of Fractions
Source: PLoS One. 2013 Nov 13;8(11):e80016. doi: 10.1371/journal.pone.0080016 (PMC3827422; doi:10.1371/journal.pone.0080016)
Supplement: Table S1 — Stimuli used in the experimental tasks. (DOCX) [file pone.0080016.s002.docx]

Table S1: Stimuli used in the experimental tasks

| **Condition** | **Fraction 1** | **Fraction 2** |
| --- | --- | --- |
| Different | 1/2 | 6/7 |
| Different | 1/2 | 2/7 |
| Different | 1/2 | 1/5 |
| Different | 1/3 | 1/8 |
| Different | 1/3 | 6/8 |
| Different | 1/3 | 2/3 |
| Different | 1/3 | 4/8 |
| Different | 2/3 | 2/8 |
| Different | 2/3 | 5/6 |
| Different | 2/3 | 2/4 |
| Different | 2/3 | 2/6 |
| Different | 1/4 | 4/5 |
| Different | 1/4 | 6/7 |
| Different | 1/4 | 4/9 |
| Different | 1/4 | 1/9 |
| Different | 1/4 | 6/9 |
| Different | 2/4 | 3/9 |
| Different | 2/4 | 2/7 |
| Different | 2/4 | 5/9 |
| Different | 3/4 | 3/5 |
| Different | 3/4 | 2/4 |
| Different | 3/4 | 2/3 |
| Different | 3/4 | 8/9 |
| Different | 1/5 | 6/8 |
| Different | 2/5 | 7/8 |
| Different | 2/5 | 3/8 |
| Different | 2/5 | 1/2 |
| Different | 3/5 | 3/8 |
| Different | 3/5 | 6/8 |
| Different | 4/5 | 2/8 |
| Different | 4/5 | 1/6 |
| Different | 4/5 | 1/2 |
| Different | 4/5 | 3/4 |
| Different | 4/5 | 4/6 |
| Different | 1/6 | 6/9 |
| Different | 1/6 | 6/7 |
| Different | 1/6 | 4/7 |
| Different | 2/6 | 7/9 |
| Different | 4/6 | 3/5 |
| Different | 4/6 | 5/6 |
| Different | 4/6 | 4/9 |
| Different | 4/6 | 7/9 |
| Different | 5/6 | 2/6 |
| Different | 5/6 | 5/9 |
| Different | 1/7 | 1/2 |
| Different | 1/7 | 1/6 |
| Different | 1/7 | 6/8 |
| Different | 2/7 | 1/4 |
| Different | 2/7 | 1/6 |
| Different | 3/7 | 4/7 |
| Different | 4/7 | 5/8 |
| Different | 4/7 | 1/2 |
| Different | 5/7 | 2/8 |
| Different | 5/7 | 2/7 |
| Different | 5/7 | 5/8 |
| Different | 5/7 | 2/4 |
| Different | 6/7 | 1/8 |
| Different | 6/7 | 1/7 |
| Different | 6/7 | 3/8 |
| Different | 1/8 | 6/8 |
| Different | 1/8 | 4/8 |
| Different | 2/8 | 7/8 |
| Different | 2/8 | 1/3 |
| Different | 2/8 | 7/9 |
| Different | 3/8 | 2/3 |
| Different | 4/8 | 7/8 |
| Different | 5/8 | 2/3 |
| Different | 5/8 | 6/8 |
| Different | 7/8 | 6/8 |
| Different | 7/8 | 7/9 |
| Different | 7/8 | 4/9 |
| Different | 7/8 | 2/9 |
| Different | 7/8 | 2/3 |
| Different | 1/9 | 6/8 |
| Different | 2/9 | 3/4 |
| Different | 2/9 | 1/4 |
| Different | 2/9 | 2/4 |
| Different | 3/9 | 8/9 |
| Different | 3/9 | 6/9 |
| Different | 3/9 | 3/4 |
| Different | 4/9 | 3/6 |
| Different | 5/9 | 2/6 |
| Different | 6/9 | 1/9 |
| Different | 6/9 | 5/6 |
| Different | 6/9 | 3/4 |
| Different | 7/9 | 2/9 |
| Different | 7/9 | 2/4 |
| Different | 8/9 | 3/6 |
| Different | 8/9 | 7/8 |
| Different | 1/5 | 4/5 |
| Different | 2/3 | 3/6 |
| Equivalent | 1/2 | 2/4 |
| Equivalent | 1/2 | 4/8 |
| Equivalent | 1/2 | 3/6 |
| Equivalent | 1/3 | 2/6 |
| Equivalent | 1/3 | 3/9 |
| Equivalent | 2/3 | 6/9 |
| Equivalent | 2/3 | 4/6 |
| Equivalent | 1/4 | 2/8 |
| Equivalent | 2/4 | 1/2 |
| Equivalent | 2/4 | 3/6 |
| Equivalent | 2/4 | 4/8 |
| Equivalent | 3/4 | 6/8 |
| Equivalent | 2/6 | 1/3 |
| Equivalent | 2/6 | 3/9 |
| Equivalent | 3/6 | 4/8 |
| Equivalent | 3/6 | 2/4 |
| Equivalent | 3/6 | 1/2 |
| Equivalent | 4/6 | 2/3 |
| Equivalent | 4/6 | 6/9 |
| Equivalent | 2/8 | 1/4 |
| Equivalent | 4/8 | 2/4 |
| Equivalent | 4/8 | 3/6 |
| Equivalent | 4/8 | 1/2 |
| Equivalent | 6/8 | 3/4 |
| Equivalent | 3/9 | 1/3 |
| Equivalent | 3/9 | 2/6 |
| Equivalent | 6/9 | 4/6 |
| Equivalent | 6/9 | 2/3 |
| Same | 1/2 | 1/2 |
| Same | 1/3 | 1/3 |
| Same | 2/3 | 2/3 |
| Same | 3/4 | 3/4 |
| Same | 1/5 | 1/5 |
| Same | 3/5 | 3/5 |
| Same | 1/6 | 1/6 |
| Same | 3/6 | 3/6 |
| Same | 1/7 | 1/7 |
| Same | 3/7 | 3/7 |
| Same | 5/7 | 5/7 |
| Same | 2/8 | 2/8 |
| Same | 3/8 | 3/8 |
| Same | 7/8 | 7/8 |
| Same | 2/9 | 2/9 |
| Same | 6/9 | 6/9 |
